# An Improved Shape from Focus Method for Measurement of Three-Dimensional Features of Fuel Nozzles

**DOI:** 10.3390/s23010265

**Published:** 2022-12-27

**Authors:** Liang Hou, Jiahao Zou, Wei Zhang, Yun Chen, Wen Shao, Yuan Li, Shuyuan Chen

**Affiliations:** 1 Department of Mechanical and Electrical Engineering, Xiamen University, Xiamen 361005, China; 2 State Key Laboratory of High-Performance Complex Manufacturing, Central South University, Changsha 410083, China; 3 Aero Engine Corporation of China (AECC) Guizhou Liyang Aviation Power Co., Ltd., Guiyang 550014, China

**Keywords:** shape from focus, three-dimensional measurement, fuel nozzles, cone, swirl slot

## Abstract

The precise three-dimensional measurement of fuel nozzles is of great significance to assess the manufacturing accuracy and improve the spray and atomization performance. This paper proposes an improved fast shape from focus (SFF) method for three-dimensional measurement of key features of fuel nozzles. In order to ensure the measurement accuracy and efficiency of the SFF, the dispersion of the measured points from a standard flat plane was used to select the optimal combination of the focus measure operator, window size and sampling step size. In addition, an approximate method for the focus measure interval is proposed to improve the measurement efficiency, which uses the peak region of the central pixel to replace the peak region of other pixels. The results show that the proposed method decreased the average computation time of the focus measure by 79.19% for the cone section and by 38.30% for the swirl slot. Compared with a reference laser scanning microscope, the measurement error in length is within 10 μm and the error in angle is within a maximum 0.15°.

## 1. Introduction

Fuel nozzles are important for aero-engines, and their dimensional accuracy determines the spray and atomization characteristics, thus affecting the performance of the whole aero-engine. The geometrical features, such as the cone and swirl slot of the nozzle, significantly affect the atomization characteristics [1,2,3,4]. As those geometrical features usually consist of small curved surfaces and require high manufacturing precision, two-dimensional measurement techniques are not applicable to them and three-dimensional (3D) measurement methods are therefore required.

Recently, 3D measurement methods have played an important role in manufacturing, health care and agricultural industries, and are mainly used to obtain 3D models of measured objects for reverse engineering, quality inspection, and dimensional measurement [5,6,7,8]. 3D measurement methods can be divided into contact and non-contact measurement methods. The typical contact measurement is probing inspection in the coordinate measuring machine (CMM). The CMM uses the probe to contact the measured object at specific points, records the measured coordinates of the contact points and extracts 3D key parameters of the object. The results from contact measurement are precise and stable [9,10], but such a measurement method is usually slow and unable to measure objects with low hardness due to surface scratches. In non-contact measurement, there are active and passive measurement methods. The terahertz-time of flight system, digital holographic interferometry and structured light, which are typical active methods, have fast measurement speeds and high precision [11,12,13]. However, active methods require extra devices to generate specific signals, increasing the complexity and cost of the equipment. Moreover, the environmental effect on the specific signals cannot be ignored in active methods. Passive methods contain mainly the stereo vision method and the monocular vision method. The stereo vision method is simple and efficient, but the accuracy is relatively low [14].

The shape from focus (SFF) [15] is a popular passive monocular vision method with advantages of high measurement accuracy, speed and low cost and has been applied to roughness measurement [16], material structure analysis in mineralogical [17], and surface topography measurement [18]. The SFF depends on accurate focus measurement that is sensitive to the focus measure operator, window size and sampling step size [19]. A comparative analysis of the focus measure operators by Pertuz et al. [20] shows that it is difficult to predict the optimal focus measure operator in the investigated optical environment. Therefore, a specific SFF measurement device must be calibrated to choose the optimal focus measure operator.

The performance of the optimal focus measure operator under different window sizes and sampling step sizes may vary. For example, during the measurement of curved or sloping objects, a large window size causes over smoothing of the object slope and more likely removes the edges, whereas a small window size is easily affected by noise [21,22,23]. In most studies, the optimal window size is selected from 3 × 3 to 29 × 29 [20,24]. Li et al. [25] proposed an adaptive selection method for the window size using multiple iterations, which resulted in a long computation time. Muhammad et al. [26] formulated the window size criteria for SFF systems with the imaging device parameters, but the formulas ignored that different focus measure operators have different performance depending on window size variation. Similarly, the optimal sampling step size is also related to the imaging device parameters, as well as the mechanical noise caused by the motion platform and environments. A small sampling step size increases the number of images in the SFF, resulting in increased computation time and memory usage, whereas a large step size decreases the measurement accuracy [27]. Simulated geometries with manual noises are used to quantitatively assess the performance of the SFF and select the suitable combination of the focus measure operator, window size and sampling step size [28,29,30].However, it is difficult to mimic the complex and real measurement environment using simulated geometries. Therefore, a proper criterion for selection of the focus measure operator with the optimized window size and sampling step size is necessary.

If an object measured by SFF has a large depth, the number of images will increase, resulting increased GPU computation time. Parallel computing significantly speeds up the computation [31], but adds an expensive cost for hardware improvement. Therefore, Gladines et al. [32] obtained an approximate depth of the measured object in advance using a fast 3D profiling technique (such as laser triangulation), in order to improve the measurement speed. It is noted that this method also used extra hardware. The fuel nozzles usually have features with large depths, especially the swirl slot feature, which causes a large computation time increase. Hence, to realize the fast 3D measurement of fuel nozzles, an accurate focus measurement method in the SFF without introducing extra hardware cost is required.

This paper proposes a fast shape from focus method for 3D measurement of key features of the fuel nozzles. First, a selection criterion for the optimized combination of the focus measure operator, window size and sampling step size based on the flatness measurement is proposed to improve the measurement accuracy. Second, to avoid large computation time for features with large depth, an approximate method for the focus measure interval is proposed to speed up the evaluation of the focus measure. Finally, the reliability of the proposed method is compared with the results from a reference laser scanning microscope.

## 2. Methodology

### 2.1. Shape from Focus

**Figure 1:**
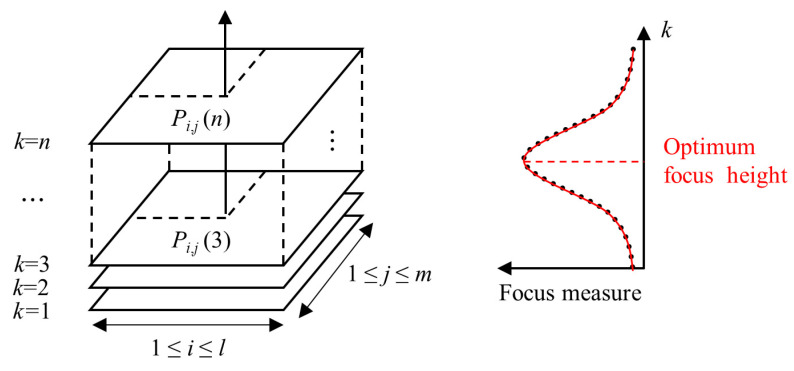
Principle of shape from focus.

The basic principle of SFF is to select the sharpest pixel from the image sequence and along the optical axis to obtain the height matrix of the measured object surface [28,33]. In practice, the image sequence is acquired by changing the camera height from the measured object with an equal sampling step. According to the imaging principle [34], the sharpest areas of the imaged object surface at different heights should appear in the corresponding order of the image sequence. Therefore, pixels at the same 2D *x*-*y* position have different degrees of sharpness in the image sequence. The height of the sharpest pixel in the image sequence can be determined by calculating and comparing the degree of sharpness of the pixels at the same *x*-*y* position over the image sequence in the *z* height direction (Figure 1). Finally, the height matrix of the measured object surface can be obtained by combining the *x*-*y* position information with the *z* height information of all the sharpest pixels in the image sequence.

Specifically, by changing the camera height, an image sequence with an equal sampling step is obtained. Each pixel in the image sequence is represented by *P_i_*_,*j*_(*k*). *i*, *j* indicate the *x* and *y* position of the pixel, respectively, and *k* is the image frame number in the *z* direction. The focus measure operator measures the sharpness of the pixel [35]

(1) $$T_{i,j}\left(k\right)=FM\left(P_{i,j}\left(k\right)\right)$$

*FM* is a focus measure operator used to calculate the focus measure *T_i_*_,*j*_(*k*) of the pixel *P_i_*_,*j*_(*k*). *T_i_*_,*j*_, for the specific pixel at the position (*i*, *j*), forms a focus curve along the height direction. It should be pointed out that such a focus curve is discrete due to the discretely obtained image sequence. This leads to the loss of information between two adjacent image frames, so the sharpest image may not have the definitively focused status [36,37]. As a result, the optimum focus heights for the most image points are difficult to calculate accurately. In order to solve this problem, approximation techniques are applied to SFF. The optimum focus height is obtained by fitting the focus curve to improve the measurement accuracy.

**Figure 2:**
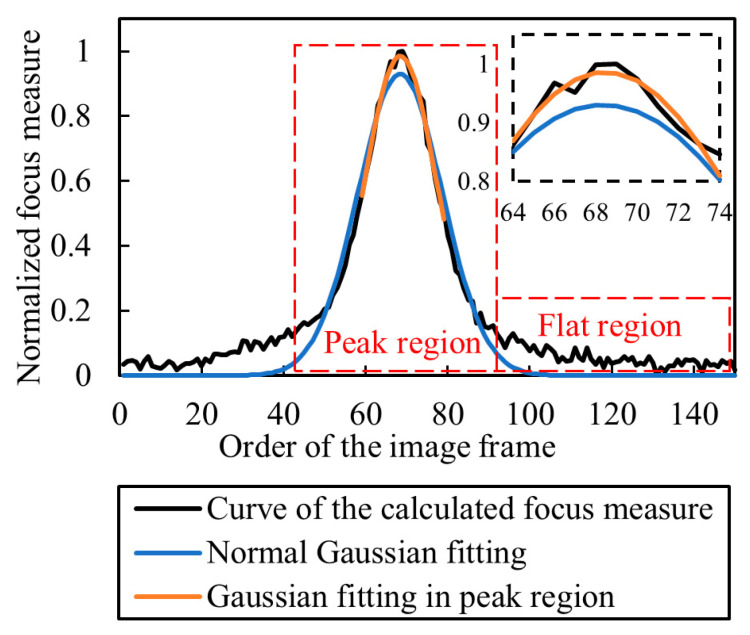
Curve fitting results of different fitting methods.

In the literature, Gaussian fitting is used to find the optimum focus height [15,20,38]. The focus curve typically has both flat and peak regions [39]. The flat region reduces the accuracy of Gaussian fitting. As the optimum focus height is determined by the peak region of the focus curve [18], Gaussian fitting taking into consideration only the peak region can avoid the influence of the flat region and improve the fitting efficiency, and the half-peak width of the focus curve is used as the criterion to get the peak region. Figure 2 shows that the fitted Gaussian curve which considers the peak region is closer to the focus curve than the traditional Gaussian fitting method over all regions.

Obviously, the key to SFF is to find the optimum focus height for each pixel in the image sequence precisely and quickly. The focus measure operator, window size and sampling step size affect the measurement accuracy and efficiency [20,24,27]. Since Gaussian fitting is only carried out in the peak region, the computation of the focus measure outside the peak region wastes a lot of time. Therefore, this paper studies the selection of the focus measure operator, window size and sampling step size to improve accuracy as well as shorten the computation time. A method for selecting the interval of the focus measure is proposed, which significantly shortens the computation time by giving up the computation of the focus measure outside the peak region.

### 2.2. Sensitivity Analysis

The performance of different combinations of focus measure operators and window sizes cannot be predicted for a particular imaging device. Therefore, it is necessary to evaluate the performance of different combinations of focus measure operators and window sizes for the given experimental platform and select the optimal combination. There is no uniform and accurate quantitative evaluation method for the SFF measurement error of a real object, and the selection of a high-performance focus measure operator and window size requires accurate identification of the optimum focus height. Therefore, narrowly-dispersed points for an ideal plane or a plane with a very small flatness indicate a good performance of the focus measure operator and window size.

For this reason, the present paper selects the dispersion of points extracted from a standard plane with a very small flatness to conduct the sensitivity analysis. To quantify the dispersion of points, the root mean square error (RMSE) of the standard plane is used. In addition, RMSEs from the proposed method and the data of Keyence VK-X1000 3D laser scanning microscope are compared to choose the final focus measure operator and window size. Pertuz et al. [20] comprehensively evaluated the performance of 36 focus measure operators. Based on the existing experimental platform and workpiece, this paper evaluates the performance of the above 36 operators and selects the final 6 operators with better performance for further evaluation. The window size selection is 5 × 5 to 29 × 29 pixels from the literature and our own experience. The abbreviations and equations for focus measure operators are shown in Table 1.

The sampling step size is also one of the most important parameters affecting the accuracy of measurement. Selection of a small step size is a conservative strategy that can guarantee the accuracy of the measurements. However, the resolution of the image sequence in the *z* direction is limited, and infinitely reducing the step size cannot improve the measurement accuracy. Moreover, a small sampling step can lead to a great increase in the number of images, resulting in heavy computation loads. Therefore, sensitivity analysis of the sampling step size is required to select the least number of images with acceptable measurement accuracy. Tang et al. [18] used a hardware system similar to this paper, and the results showed that the average measurement errors for the two sampling steps, 5 μm and 10 μm, were both around 5 μm. In addition, Muhammad et al. [27] presented a method for choosing the sampling step size, and the recommended step size of the measurement system in this work is about 24 μm. Although this method does not consider the effect of the mechanical noise on the step size, it is still selected as the upper limit of the step size for assessment. Hence, this paper selects 5–30 μm step sizes for sensitivity analysis and the minimal RMSE for a standard plane is used to select the final step size.

**Figure 3:**
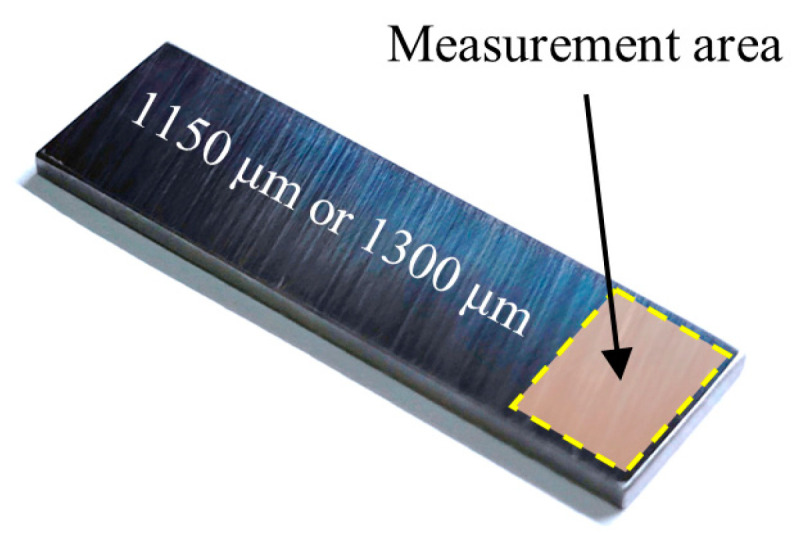
Standard gauge block for pre-experimentations.

In order to select the focus measure operator, window size and sampling step size, this paper sets up two pre-experiments. Standard gauge blocks (Figure 3) with a thickness of 1150 μm or 1300 μm (flatness less than 0.08 μm) were used in the pre-experiment as the standard plane. In addition, the RMSE of the plane obtained by this method is compared with the measurement results from the laser scanning microscope to assess the measurement accuracy.

### 2.3. Determination of the Approximate Focus Measure Interval

The traditional SFF has to calculate the focus measure for a single pixel *P_i_*_,*j*_ at the position (*i*, *j*) on all image frames to form a complete focus curve. When Gaussian fitting only considers the peak region, the flat region does not affect the determination of the optimum focusing height. Therefore, the calculation of the focus measure in the flat region is unnecessary, which reduces the calculation efficiency.

**Figure 4:**
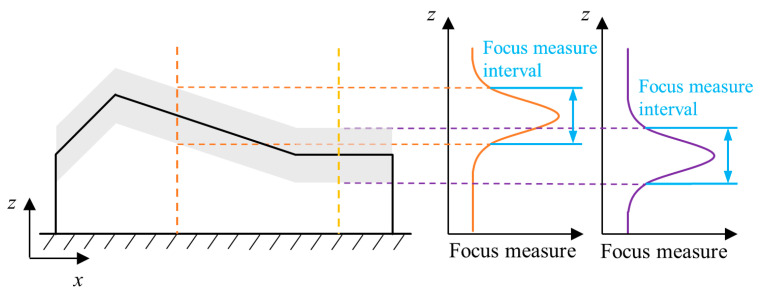
Measured object model and ideal focus measure interval.

**Figure 5:**
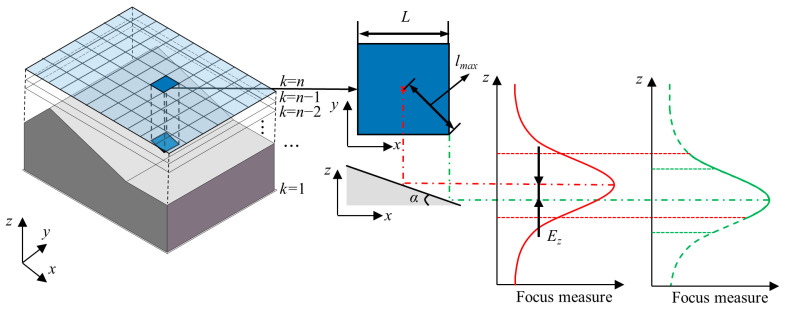
Approximate focus measure interval.

For a typical object (Figure 4) whose surface contains both flat and inclined features with different slope angles, only the focus measure of the shadow area (corresponding to the peak region of the focus curve) on the surface in the height direction is valuable. The computation time can be reduced by limiting the focus measure interval to this region. Therefore, this paper proposes a method for reduction of the focus measure interval. Figure 5 shows the principle of this method. In the *x*-*y* plane, all images over the whole image sequence are divided into the same number of square areas, and the peak region of the central pixel position of each square is taken to approximately replace the peak region of all pixel positions in it. Therefore, in a single square, the complete focus curve only needs to be calculated in the central pixel position, and other pixel positions just need the calculation of the focus measure in the peak area.

The proposed method approximates the peak region at the other pixel positions, which may generate errors during determination of the optimal focus height. The errors depend on the slope of the feature to be measured. For flat features, the peak regions of the focus curves at different pixel positions in a specific square area are the same, so the proposed method can be perfectly applied to this feature. However, for slope features, the peak regions of the focus curves at different pixel positions in the square area are different, which leads to a deviation *E_z_* from the peak region for the slope feature as shown in Figure 5. Therefore, the length of the side of the square should be carefully selected before application of the proposed method.

*E_z_* can be considered proportional to the distance *l* between the pixel position and the center of the square and reaches the maximum value when the distance *l* is maximum *l_max_*. Moreover, it is also related to the slope of the measured feature. The increase in the slope angle induces an increase of *E_z_* at the same position. The deviation of the peak region causes errors in determination of the optimum focus height from the Gaussian fitting. The relationship between the two depends on the characteristics of the focus curve, as well as the experimental platform. In general cases, according to the surface slope of the measured object, the maximum allowable slope *α_max_* is obtained. In order to use this method in as many areas as possible and to divide as few squares as possible (that is, to calculate the pixel position of the complete focus curve as little as possible), *α_max_* should be as small as possible while being larger than the slope of most areas (sometimes this is difficult to choose). Therefore, it is necessary to determine the most suitable length *L* of the side of the square according to the maximum slope angle *α_max_* and the maximum allowable deviation *E_zmax_*. The relationship among *L*, *α_max_* and *E_zmax_* is expressed as

(2) $$L=\sqrt{2}l_{\max}=\frac{\sqrt{2}E_{z\max}}{\tan\alpha_{\max}}$$

Since some areas with large slopes are abandoned when *α_max_* is selected, when the slope between the centers of two adjacent squares is bigger than *α_max_*, this method is disabled in the corresponding area. This method can shorten the computation time of SFF, especially when the dimension of the image sequence is large.

### 2.4. Dimension Extraction

The geometrical characteristics of the dual-orifice pressure-swirl nozzle significantly affect the flow performance [1,2]. These geometrical characteristics are mainly cones and swirl slots. Therefore, 3D dimension extraction methods are required to characterize those key geometric dimensions. For cones, the semi-vertical angle needs to be measured, and the width, height and helix angle of the swirl slot should be measured. Therefore, different dimension extraction methods are required to extract the key dimensions of different geometrical features.

#### 2.4.1. Semi-Vertical Angle of the Cone

**Figure 6:**
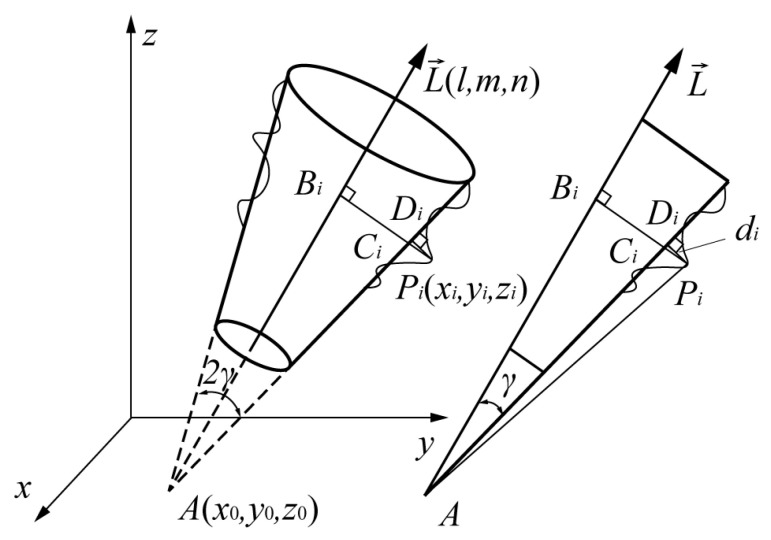
Cone in 3D coordinate system.

The extraction of the semi-vertical angle is obtained by point cloud fitting and the minimum zone evaluation method based on particle swarm optimization (PSO) is used for fitting. An ideal cone can be expressed by three parameters: cone apex *A*(*x*_0_, *y*_0_, *z*_0_), semi-vertical angle *γ* and axis vector $\vec{L}$(*l*, *m*, *n*), as shown in Figure 6. The axis vector can be expressed as [40]

(3) $$\frac{x-x_{0}}{l}=\frac{y-y_{0}}{m}=\frac{z-z_{0}}{n}$$

which can be rewritten as

(4) $$\frac{x-x_{0}}{q_{1}}=\frac{y-y_{0}}{q_{2}}=z-z_{0}$$

where *q*_1_ = *l*/*n*, *q*_2_ = *m/n*. The distance *d_i_* between any point *P_i_*(*x_i_, y_i_, z_i_*) in the point cloud and the ideal conical surface is defined

(5) $$\begin{array}{cc} d_{i} & =\left|P_{i}D_{i}\right|=\left(\left|B_{i}P_{i}\right|-\left|B_{i}C_{i}\right|\right)\cos\gamma \\ & =\left(\left|B_{i}P_{i}\right|-\left|AB_{i}\right|\tan\gamma \right)\cos\gamma \\ & =\left|B_{i}P_{i}\right|\cos\gamma -\left|AB_{i}\right|\sin\gamma \end{array}$$

where

(6) $$\left\{ \begin{array}{l} \left|P_{i}A\right|=\sqrt{{\left(x_{0}-x_{i}\right)}^{2}+{\left(y_{0}-y_{i}\right)}^{2}+{\left(z_{0}-z_{i}\right)}^{2}}\\ \left|AB_{i}\right|=\frac{\left|q_{1}\left(x_{0}-x_{i}\right)+q_{2}\left(y_{0}-y_{i}\right)+z_{0}-z_{i}\right|}{\sqrt{q_{1}^{2}+q_{2}^{2}+1}}\\ \left|B_{i}P_{i}\right|=\sqrt{{\left|P_{i}A\right|}^{2}-{\left|AB_{i}\right|}^{2}} \end{array}\right.$$

It can be seen that *d_i_* is a function of variables (*x*_0_, *y*_0_, *z*_0_, *q*_1_, *q*_2_). Therefore, the objective function of PSO is

(7) $$f\left(x_{0},y_{0},z_{0},q_{1},q_{2},\gamma \right)=\min\left(\max(d_{i})-\min\left(d_{i}\right)\right)$$

The cone dimensions extracted from the point cloud can be obtained through minimizing Equation (7).

#### 2.4.2. Dimensions of the Swirl Slot

**Figure 7:**
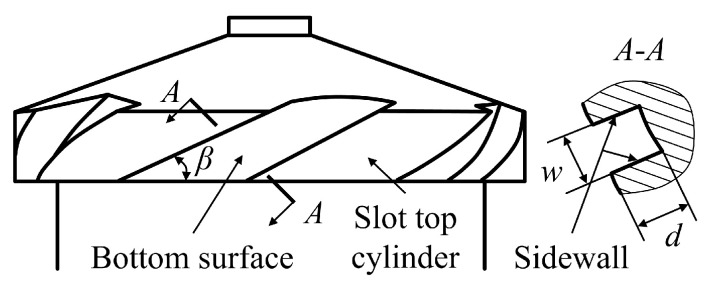
Swirl slot of the inner part of the nozzle.

**Figure 8:**
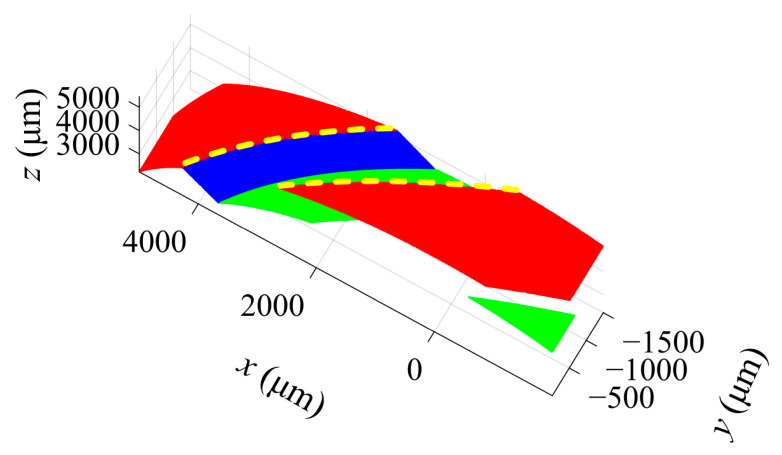
Point cloud segmentation of the swirl slot.

The swirl slot is composed of the top, bottom and side surfaces. The top and bottom surfaces are cylindrical surfaces; the side surface is helical, as shown in Figure 7. The key dimensions of the slot are the depth *d*, helix angle *β* and width *w*. Figure 8 divides the obtained point clouds on the slot surfaces into three parts, i.e., top (red), bottom (green) and side (blue). Cylinder fitting is applied to the top point cloud and the bottom point cloud. The cylinder fitting method is a special type of cone fitting with *γ* equal to 0, as in Section 2.4.1 [40]. The difference between the radii of the two cylinders obtained by fitting is the slot depth *d*. To obtain *β* and *w*, the whole point cloud is mapped to a Cartesian coordinate system according to the following relationship [41]

(8) $$\{\begin{array}{l} {x_{i}}^{\prime }=\sqrt{x_{i}^{2}+y_{i}^{2}}\\ {y_{i}}^{\prime }=\sqrt{x_{i}^{2}+y_{i}^{2}}\cdot \arctan\frac{y_{i}}{x_{i}}\\ {z_{i}}^{\prime }=z_{i} \end{array}$$

**Figure 9:**
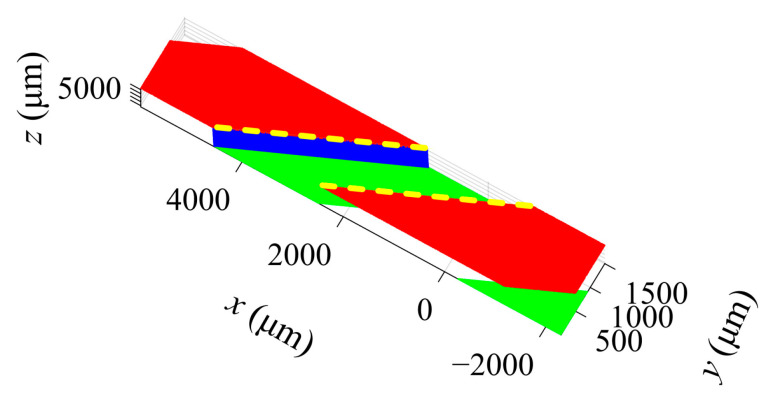
Mapped to Cartesian coordinate system.

The mapped top and side surfaces are perpendicular, as shown in Figure 9. Planar fitting is conducted to the mapped side surface, and the helix angle *β* is the angle between the fitted plane and the *y* axis. The width *w* is extracted from the distance between the two edges as highlighted in Figure 9.

## 3. Experimental Verification

### 3.1. Experimental Setup

**Figure 10:**
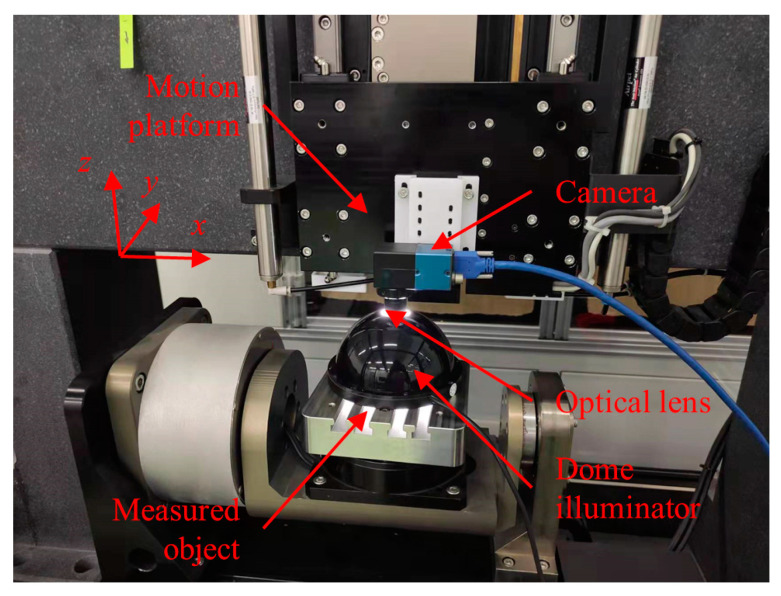
Shape from focus measurement system.

In order to verify the proposed three-dimensional measurement method using SFF, an experimental platform was established as shown in Figure 10. The system mainly comprises a camera (Daheng Image Beijing, China, MER2-1220-32U3C-W90), a telecentric lens (Edmund Optics Shenzhen, China 1 × 40 mm WD CompactTL™ Telecentric Lens), a dome light source and a motion platform. The motion platform has a worktable, and a *z*-axis motion module driven by a linear motor and control module (Beckhoff CX2020). The camera is installed on the *z*-axis motion module in order to ensure the lens optical axis is parallel to the *z*-axis direction. The measured object is placed on the worktable, and the light source is placed on the top of the measured object. Detailed parameters for the experimental platform are shown in Table 2.

**Figure 11:**
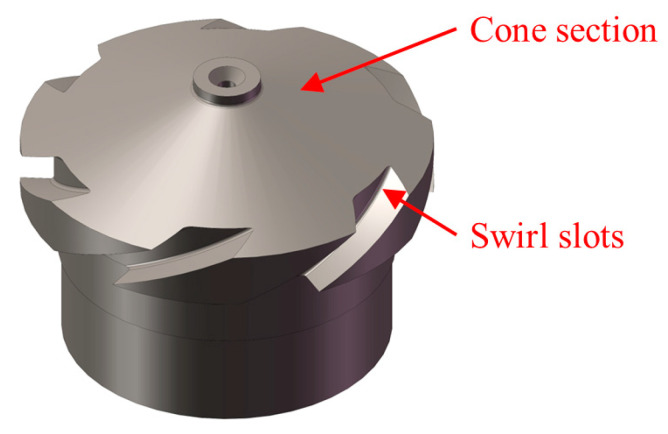
Inner part of a dual-orifice pressure-swirl nozzle.

**Figure 12:**
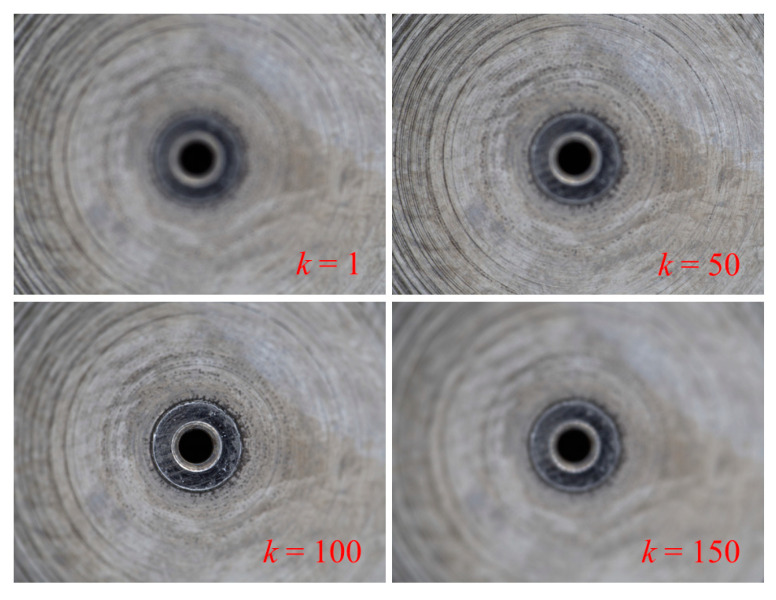
Sharpness variation of an image sequence.

In this paper, the inner part of dual-orifice pressure-swirl nozzle is measured, as shown in Figure 11. The nozzle is made of 9Cr18, the manufacturing process is the cone section being processed by turning, and the swirl slots are processed by milling. Before measurement, the camera is moved downward and approaches the nozzle until the image is completely blurred. This position is set as the initial position, and the corresponding image frame number is *k* = 1. The camera begins to move upward with the optimal sampling step size obtained from the sensitivity analysis, and captures an image at every sampling step until the image is completely blurred again. Figure 12 shows several sample images of the image sequence, which shows a trend from completely blurred to partially blurred, and again completely blurred. All computations of image sequence are run on a PC with Intel i7 10700F processor and 16 GB RAM in Matlab R2021a.

### 3.2. Results from Sensitivity Analysis

**Figure 13:**
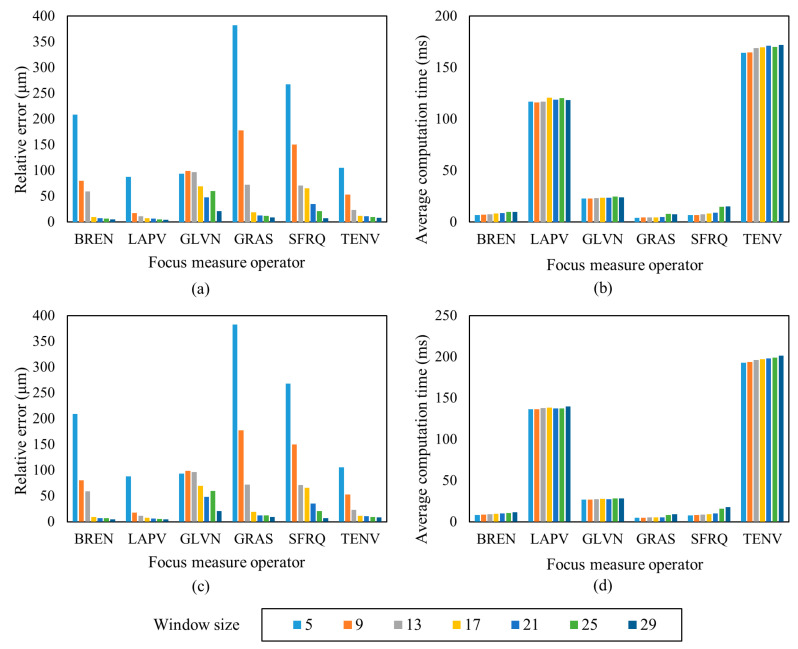
Standard gauge block plane measurement with different focus measure operators and window sizes. (**a**) Measurement error, 1150 μm standard gauge block; (**b**) Computation time, 1150 μm standard gauge block; (**c**) Measurement error, 1300 μm standard gauge block; (**d**) Computation time, 1300 μm standard gauge block.

According to the proposed selection method for the focus measure operator and window size, the standard gauge blocks are measured and RMSE is extracted from the fitted plane. The best focus measure operator and window size are chosen if the difference of the RMSEs extracted from the proposed method and the laser scanning microscope measurement data is small. The corresponding difference of the RMSEs and the computation time of the proposed method are shown in Figure 13. The variations of the differences of the RMSEs of the two measured standard gauge blocks (1150 μm or 1300 μm) are similar, implying good repeatability of the proposed method. The difference of the RMSEs of each operator decreases with the increase of the window size, and the computation time increases with the rise in the window size. When the window size is small, the operators have small differences, such as with LAPV and TENV, often needing a long computation time.

According to the measurement accuracy requirements of fuel nozzles, the measurement error should be within 10 μm. By taking into consideration the difference of the RMSEs, computation time and also the over smoothing due to large window sizes [24], the BREN operator with a window size of 21 × 21 (pixels) is finally selected for measurement. In this case, the differences of the RMSEs of the two standard gauge blocks are 7.20 μm and 6.21 μm, respectively, and the average computation time for each pixel position is about 10 ms, which has a high computation efficiency and meets the measurement requirements for the fuel nozzle.

**Figure 14:**
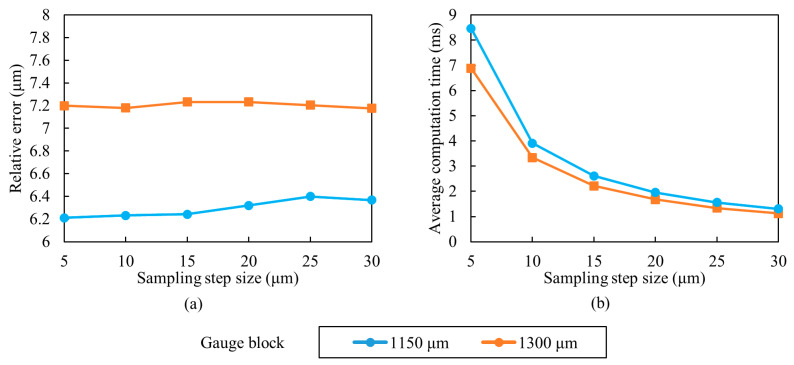
Standard gauge block plane measurement with different sampling step sizes. (**a**) Measurement error and (**b**) Computation time.

To determine the optimal sampling step size, two set of images of the standard gauge blocks of 1150 μm and 1300 μm were obtained with the same basic sampling step size of 5 μm. Other step sizes from 10 μm to 30 μm were sampled from the basic step size of 5 μm. The selected focus measure operator and window size in the above context are used for selection of the step size. The difference of the RMSEs extracted from the proposed method and the laser scanning microscope measurement data is also used as the index for measurement accuracy. The difference of the RMSEs and computation time are shown in Figure 14. It can be seen that the difference decreases gradually with the step size, but the difference among the different step sizes is minimal. This is Gaussian fitting that reduces the error generated by larger sampling steps. A 20 μm step size is finally selected for measurement by considering the measurement accuracy and computation complexity. Under this sampling step, the differences of the RMSEs for the two standard gauge blocks are 7.23 μm and 6.32 μm, respectively, and the average computation time for each pixel position is shortened from about 10 ms to about 2 ms compared with the 5 μm sampling step.

### 3.3. Approximate Focus Measure Intervals

**Figure 15:**
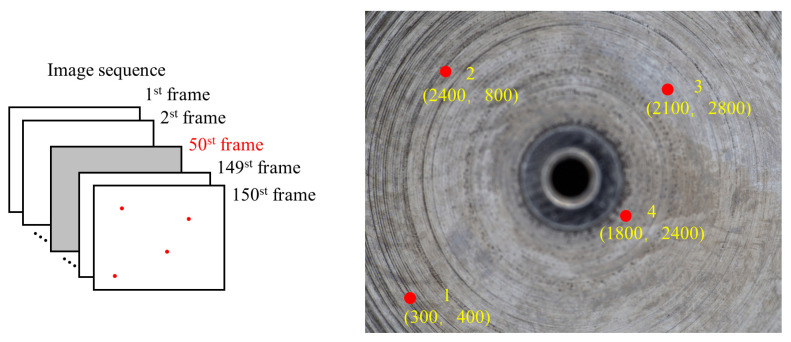
Position of image points 1–4.

In order to get the optimal focus measure interval, pre-experiments were carried out. Firstly, in order to prevent error accumulation, the error of the optimum focus height caused by the peak region deviation is set to be under 1 μm. The relationship between the error of the optimum focus height and the peak region deviation depends on the characteristics of the focus curve. Positions of the 4 pixels were randomly selected in Figure 15 to empirically obtain such a relationship. By analyzing the obtained errors of the optimum focus height and the peak region deviations *E_z_* in the four focus curves, it is found that when *E_z_* ≤ 40 μm, the errors of the optimum focus heights of the four pixels are all less than 1 μm, so we take *E_z__max_* = 40 μm. In addition, the slope angle of the interested surfaces of the measured object is mostly below 30°, and the corresponding *L* is 97.98 μm (52 pixels) according to Section 2.3. For the convenience of practical operation, the side length *L* of the square is approximated to 51 pixels (*L* = 94.35 μm).

### 3.4. Fuel Nozzle Measurement

**Figure 16:**
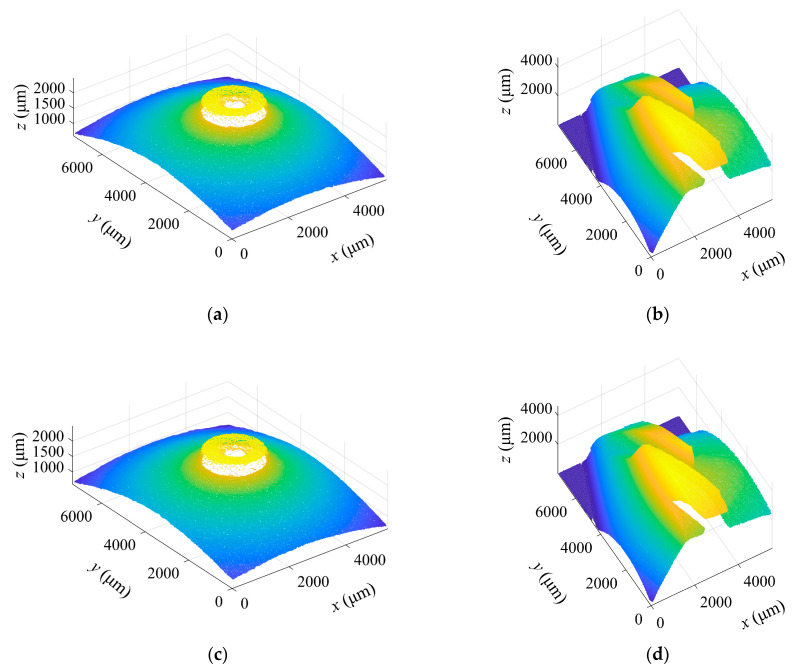

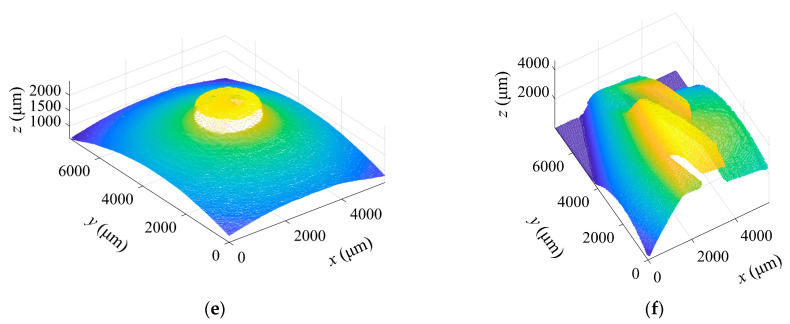
Point clouds from the different methods. (**a**,**b**) are measured by the traditional SFF; (**c**,**d**) are measured by the proposed SFF; and (**e**,**f**) are measured by an laser scanning microscope.

The inner parts of four dual-orifice pressure-swirl nozzles were measured. The camera height moved upward with a sampling step of 20 μm to obtain the image sequences for the cone and swirl slots. The BREN operator and a window size of 21 × 21 (pixels) were used for SFF, and the height matrix of the measured object was converted into a point cloud. Figure 16a,b demonstrate the point clouds obtained by traditional SFF methods.

Figure 16c,d show the point clouds obtained using the focus measure interval approximate method. The results indicate that compared with the traditional method, the average computation time of the focus measure of each pixel position for the cone is shortened from 2.431 ms to 0.506 ms, reducing computation time by 79.19% and the average error of the optimum focus height over all pixel positions is 0.26 μm. As the swirl slot has many areas with large slope angles, the effect of this method may be reduced. Therefore, compared with the traditional method, the average computation time of the focus measure of each pixel position is shortened from 6.635 ms to 4.094 ms, giving a computation time decrease of 38.30% and the average error of the optimum focus height is 0.23 μm. Obviously, the proposed method has a minimal impact on the measurement accuracy and significantly reduces the computation time.

Finally, the dimension extraction method for the cone in Section 2.4.1 is used to fit the point cloud of the cone, and the measured semi-vertical angle is shown in Table 3. After the point cloud segmentation, cylindrical fitting and cylindrical expansion are performed to measure the depth, width and helix angle of the swirl slot. The measurement results are shown in Table 4. The point clouds obtained using the laser scanning microscope are shown in Figure 16e,f, which are also processed using the proposed method for dimension extraction.

Compared with the measurement results from the laser scanning microscope, the proposed method based on SFF gives a semi-vertical angle measurement error of less than 0.1° for the cone. The measurement error of the slot depth and width is less than 10 μm, and the measurement error of the slot helix angle is less than 0.15°. Therefore, the proposed method has an acceptable measurement accuracy and is suitable for the three-dimensional measurement of fuel nozzles.

## 4. Conclusions

Precise three-dimensional measurement of fuel nozzles helps assess and control manufacturing accuracy, which further affects the combustion performance of aero-engines. In this paper, an improved fast SFF method is proposed to conduct a precise and fast three-dimensional measurement of key features of dual-orifice pressure-swirl nozzles. The key findings are summarized as follows.

- The experimental results show that the improved SFF method can perform three-dimensional measurements of fuel nozzles with high precision. Compared with the measurement results of the laser scanning microscope, the proposed method gives a measurement error less than 0.1° for the semi-vertical angle, 10 μm for the swirl slot depth and width, and 0.15° for the swirl slot helix angle.
- The dispersion of the measured points from a standard flat plane is used to assess the performance of different focus measure operators, window sizes and sampling step sizes. The BREN focus measure operator with a 21 × 21 (pixels) window size and a sampling step size of 20 μm is chosen for our experimental setup. The results show that the chosen parameters can balance the measurement time and accuracy.
- An approximate method for the focus measure interval is proposed, which uses the peak region of the central pixel to replace the peak region of other pixels. The experimental results show that the proposed method has decreased the average computation time of the focus measure at each pixel position by 79.19% for the cone section and by 38.30% for the swirl slot without using extra hardware.

It is pointed out that the optimal parameters of the SFF are heavily dependent on measurement devices, so the sensitivity analysis needs to be re-performed when some devices are replaced due to work requirements. Moreover, *L* for the measured features is selected based on pre-experiments and the known CAD model for the nozzle. If the CAD model is unknown, some pre-experiments may be required to determine the suitable *L*. Additionally, a fixed *L* is applied to the investigated features in the work. A large curved surface with a great variation of the slope angle may require a different *L* for different sub-surfaces. The proposed method is not currently suited for such a complex feature and will be our future research direction.

## Figures and Tables

**Table 1:** Abbreviations for different focus measure operators.

Focus Measure Operator	Abbr.	Expression
Brenner’s focus measure	BREN	$\phi_{x,y}={\sum_{\left(i,j)\in \Omega (x,y\right)}\left(I\left(i,j\right)-I\left(i+2,j\right)\right)}^{2}$
Variance of Laplacian	LAPV	$\phi_{x,y}={\sum_{\left(i,j)\in \Omega (x,y\right)}\left(\Delta I\left(i,j\right)-\bar{\Delta I}\right)}^{2}$
Gray-level local variance	GLVN	$\phi_{x,y}={\sum_{\left(i,j)\in \Omega (x,y\right)}\left(L_{v}\left(i,j\right)-\bar{L_{v}}\right)}^{2}$
Squared gradient	GRAS	$\phi_{x,y}=\sum_{\left(i,j)\in \Omega (x,y\right)}I_{x}{\left(i,j\right)}^{2}$
Spatial frequency measure	SFRQ	$\phi_{x,y}=\sqrt{\sum_{\left(i,j)\in \Omega (x,y\right)}I_{x}{\left(i,j\right)}^{2}+\sum_{\left(i,j)\in \Omega (x,y\right)}I_{y}{\left(i,j\right)}^{2}}$
Tenengrad variance	TENV	$\phi_{x,y}={\sum_{\left(i,j)\in \Omega (x,y\right)}\left(G_{x}\left(i,j\right)-\bar{G}\right)}^{2}$

**Table 2:** Parameters for the experimental platform.

Parameter	Value
Image size (pixel)	4024 × 3036
Pixel Size (μm)	1.85
Lens magnification	×1
Numerical aperture of lens	0.045
Repeated positioning error of motion platform (μm)	<1
Measuring ranges in the *z* direction (μm)	6000

**Table 3:** Measurement result of semi-vertical angle.

Atomizer Case	Measured by SFF (°)	Measured by Keyence (°)	Absolute Deviation (°)
A	71.9886	72.0203	0.0317
B	72.1016	72.1800	0.0784
C	72.3479	72.3889	0.0410
D	71.8998	71.8353	0.0645

**Table 4:** Measurement result of swirl slot.

Atomizer Case	Parameter Type	Measured by SFF	Measured by Keyence	Absolute Deviation
A	Depth of swirl slot *d* (μm)	854.2300	852.9883	1.2417
	Width of swirl slot *w* (μm)	909.7891	915.1784	5.3893
	Helical angle *β* (°)	22.9541	22.8403	0.1138
B	Depth of swirl slot *d* (μm)	854.9141	859.2065	4.2924
	Width of swirl slot *w* (μm)	952.5697	955.2558	2.6861
	Helical angle *β* (°)	22.8172	22.7169	0.1003
C	Depth of swirl slot *d* (μm)	882.4282	884.9443	2.5161
	Width of swirl slot *w* (μm)	919.0835	912.3630	6.7205
	Helical angle *β* (°)	22.9648	23.0911	0.1263
D	Depth of swirl slot *d* (μm)	857.7256	863.4785	5.7529
	Width of swirl slot *w* (μm)	910.1041	904.2358	5.8683
	Helical angle *β* (°)	23.3675	23.2531	0.1144

## Data Availability

Not applicable.
